# Comprehensive Analysis of Differentially Expressed lncRNAs in the Perivascular Adipose Tissue of Patients with Coronary Heart Disease

**DOI:** 10.31083/j.rcm2310341

**Published:** 2022-10-11

**Authors:** Xianwei Xie, Sunying Wang, Jingyi Rao, Jing Xue, Kaiyang Lin, Na Lin, Ke Li, Shilun Wu, Wenjia Liang, Yansong Guo

**Affiliations:** ^1^Department of Cardiology, Shengli Clinical Medical College of Fujian Medical University, Fujian Provincial Hospital, 350013 Fuzhou, Fujian, China; ^2^Beijing Tiantan Hospital, China National Clinical Research Center of Neurological Diseases, Advanced Innovation Center for Human Brain Protection, The Capital Medical University, 100070 Beijing, China; ^3^Fujian Provincial Key Laboratory of Cardiovascular Disease, Fujian Provincial Center for Geriatrics, Fujian Clinical Medical Research Center for cardiovascular diseases, 350000 Fuzhou, Fujian, China; ^4^Fujian Heart Failure Center Alliance, 350000 Fuzhou, Fujian, China; ^5^Department of Hepatobiliary Surgery, Beijing Chaoyang Hospital Affiliated to Capital Medical University, 100043 Beijing, China

**Keywords:** coronary heart disease, Atherosclerosis, perivascular adipose tissue, immune system, long non-coding RNA, competing endogenous RNA

## Abstract

**Background:**

Coronary heart disease is a highly prevalent inflammatory disease caused by coronary atherosclerosis. Numerous studies have revealed that perivascular adipose tissue is closely associated with atherosclerosis. Here, we conducted a comprehensive analysis of long non-coding RNAs and mRNAs differentially expressed in perivascular adipose tissue in patients with coronary heart disease.

**Methods:**

We conducted Gene Ontology term and Kyoto Encyclopedia of Genes and Genomes pathway enrichment analyses of the differentially expressed genes. Furthermore, single sample gene set enrichment analysis, immune infiltration analysis, and co-expression analysis of differentially expressed long non-coding RNAs and immune gene sets were performed. Finally, the starBase and miRTarBase databases were used to construct a competing endogenous RNA network.

**Results:**

The results show that aortic perivascular adipose tissue has higher inflammation and immune infiltration levels in patients with coronary heart disease. Dysregulated long non-coding RNAs may be related to immunity, inflammation, and hypoxia.

**Conclusions:**

The findings of this study provide new insights into atherosclerosis and coronary heart disease.

## 1. Introduction

Coronary heart disease (CHD), a common cardiovascular disease, is one of the major causes of death worldwide [1, 2]. Atherosclerosis (AS) is the common pathological basis of CHD, and its etiology is complex, involving endothelial cell dysfunction, immune response, oxidative stress, and other mechanisms [3, 4, 5]. Many studies in recent years have discovered that Perivascular adipose tissue (PVAT) dysfunction is implicated in the onset and progression of AS via a paracrine or endocrine pathway [6, 7]. PVAT covers the majority of blood vessels, including large arteries and veins, as well as small and resistive vessels, under physiological conditions, PVAT exhibits anti-inflammatory roles and is important in vasodilation regulation [8]. However, under pathophysiological situations such as obesity, PVAT becomes dysfunctional and promotes the infiltration of inflammatory immune cells and local oxidative stress, thereby leading to the dysfunction of the underlying vascular smooth muscle cells and endothelial cells [8, 9, 10]. Hence, dysfunctional PVAT promotes cardiovascular disease progression.

So far, many studies have found substantial variations in gene expression in PVAT at sites of AS [11, 12, 13]. Tang *et al*. [14] found that Ribosomal Protein S3A (RPS3A) expression is decreased in the epicardial adipose tissue of CHD patients and the PVAT of ${\mathrm{ApoE}}^{\text{-/-}}$ mice fed a high-fat diet, and RPS3A knockdown in the peri-aortic adipose tissue of mice accelerated vascular inflammation and AS development. Mu *et al*. [15] found that BMP4 is highly expressed in normal PVAT, while reduced in mouse and human atherosclerotic PVAT. Moreover, knockdown of BMP4 in adipocytes leads to increased production of pro-inflammatory cytokines, which trigger endothelial cell inflammation and promote AS [15]. Mazzotta *et al*. [13] compared the differences in gene expression between PVAT of the aorta, which is atherosclerosis-prone, and that of the internal mammary artery (IMA), which is atherosclerosis-resistant, and the result found that these differential genes are enriched in signaling pathways related to AS [13]. Therefore, exploring the differential genes in PVAT of CHD patients may help us deepen our understanding of CHD and AS and discover new potential therapeutic targets.

However, current studies in PVAT in the field of CHD or AS have mainly focused on genes encoding proteins, with few studies on non-coding RNAs. There is mounting evidence that non-coding RNAs play a prominent role in the development of cardiovascular diseases [16, 17, 18]. Several studies have demonstrated different expression profiles of lncRNAs during CHD [19, 20]. In this study, we analyzed the differentially expressed mRNAs and lncRNAs in patients with CHD based on the study of Mazzotta *et al*. [13]. And through the method of bioinformatics analysis to speculate the possible role of these unknown function lncRNAs in patients with CHD. This study provides new insights into AS and CHD and lays the foundation for the development of targeted therapies.

## 2. Materials and Methods

### 2.1 Data Sources and Processes

Gene Expression Omnibus (GEO) (https://www.ncbi.nlm.nih.gov/geo/) is a functional public genomic data repository. We downloaded the gene expression microarray datasets GSE152326 and GSE21545 from the GEO database. The GSE152326 dataset contains data of 10 perivascular adipose tissue samples collected from the proximal aorta and left IMA (LIMA) of 5 patients during coronary artery bypass surgery [13]. Clinical features of included CHD patients can be seen in **Supplementary Table 1**. The GSE21545 dataset contains data of 97 peripheral blood mononuclear cells (PBMC) samples collected from patients with AS [21]. Clinical features of 97 patients with AS can be seen in **Supplementary Table 2**. Datasets were annotated using Perl 5 (version 30). The probe IDs were replaced with the Entrez ID or gene symbol. The median expression level of all probes was analyzed if more than one probe corresponded to one gene. For GSE152326, we re-annotated the gene types using HGNC BioMart (https://biomart.genenames.org/) and obtained the expression profiles, including 4387 lncRNAs and 18365 protein-coding genes. Differentially expressed genes (DEGs) between the proximal aorta PVAT and LIMA PVAT were identified using the Limma package in R (version 3.6.3). The threshold for statistical significance was set as |$\log_{2}$FC| ≥ 1, and a *p* value of <0.05 indicates statistically significant DEGs.

### 2.2 Gene Functional Enrichment Analysis

Gene Ontology (GO) term functional annotation and Kyoto Encyclopedia of Genes and Genomes (KEGG) pathway enrichment analyses were conducted using the “Cluster Profiler” package in R [22]. Sangerbox 3.0 (http://vip.sangerbox.com/home.html) an online bioinformatics visualization platform for visualizing results. Results with *p *< 0.05 after applying Benjamini–Hochberg correction were considered significant. GSEA software (version 3.0) was used to identify the most significant functional terms between the proximal aorta PVAT and LIMA PVAT groups. “c2.cp.kegg.v7.4.symbols.gmt” from the Molecular Signatures Database (MSigDB) (http://www.gsea-msigdb.org/gsea/index.jsp) was used as the reference gene set [23, 24]. A gene set was regarded as significantly enriched when *p* was <0.05 and false discovery rate (FDR) was <0. 25. Cell signaling pathways were visualized using an online web tool Pathview (https://pathview.uncc.edu/) [25].

### 2.3 Evaluation of Immune Cell Infiltration

Single sample gene set enrichment analysis (ssGSEA) was performed on immune cell types in samples from the GSE152326 dataset using the GSVA R package (version 3.6.3) [26]. A meta-gene for each immune cell type was scored based on the ssGSEA score, according to the method of Zhang *et al*. [27]. Downloaded cell markers for 22 immune cells including B cells, macrophages, and natural killer cells from the cell-specific marker gene database PanglaoDB (https://panglaodb.se) [28]. The Wilcoxon test was used to evaluate the differences between the proximal aorta and LIMA artery groups. The results were visualized using Sangerbox 3.0, a free online platform for data analysis (http://vip.sangerbox.com/home.html). Results with *p *< 0.05 were considered statistically significant. The correlation between immune cell ssGSEA scores in aorta PVAT was calculated using the Pearson’s method. Sangerbox 3.0 (http://vip.sangerbox.com/home.html) was used to visualize the results, and results with *p *< 0.05 were defined as statistically significant.

### 2.4 Establishment of Immune-Related lncRNAs

Immune-related genes were retrieved from the Molecular Signatures Database (MSigDB) (http://www.gsea-msigdb.org/gsea/index.jsp), including two gene sets, immune response (M19817) and immune system process (M13664). Thereafter, immune-related lncRNAs were defined based on the correlation analysis between the mRNA expression level and lncRNA expression data (|R| >0.9, *p *< 0.01). The lncRNA–mRNA co-expression network was visualized using Cytoscape software (version 3.7.2) [29]. Pearson correlation analysis was performed to elucidate the relationship between immune-related lncRNA expression and immune-related genes expression.

### 2.5 Prognosis Analysis of Immune-Related lncRNAs

The 97 PBMC samples from GSE21545 were used for prognosis analysis as the method of Liu *et al*. [30]. The 97 patients with AS were followed for an average of 44 months, and the ischemic events were defined as myocardial infarctions or ischemic strokes. Based on the expression value of each lncRNA in the microarray, we divided the patients into two groups and examined their prognosis. The prognosis of patients in each group was estimated by Kaplan-Meier, and the comparison of survival prognosis between the two groups was performed by log-rank test. To select the optimal expression cutoff for the most significant patient group, we grouped all 20th to 80th percentile expression values, tested for significant differences in survival outcomes between groups, and derived the lowest log-rank *p* value. Sangerbox 3.0 (http://vip.sangerbox.com/home.html) was used for statistics and visualization.

### 2.6 Prediction of Subcellular Localization of the lncRNAs

We predicted the subcellular localization of other lncRNAs by using the tool lncLocator (https://www.csbio.sjtu.edu.cn/bioinf/lncLocator). lncRNA sequences were obtained from the Ensembl (http://asia.ensembl.org/index.html) or UCSC (http://genome.ucsc.edu/) database. If one lncRNA gene symbol encoded multiple transcripts, we selected the ensemble canonical transcript sequence. The final results were analyzed and presented using GraphPad Prism 8 software (version 8.0; GraphPad Software, San Diego, CA, USA).

### 2.7 Construction of a lncRNA-Associated ceRNA Network

lncRNA–miRNA interactions were predicted using starBase v2.0 database (http://starbase.sysu.edu.cn/index.php) [31]. The lncRNA–miRNA pair that satisfies CLIP-Data ≥1 and Degradome-Data ≥0 in the starBase database was selected as the candidate pair. miRNA target genes were then acquired using the miRTarBase database (http://miRTarBase.cuhk.edu.cn/) [32]. The miRNA–mRNA pairs with experimental reports (such as the reporter assay) included in the miRTarBase database were selected as candidate miRNA–mRNA pairs. Finally, among these predicted miRNA–lncRNA and miRNA–mRNA pairs, we selected lncRNAs and mRNAs differentially expressed in aortic PVAT to construct the subsequent competing endogenous RNA (ceRNA) networks. The lncRNA–miRNA–mRNA network was generated using Sangerbox 3.0 (http://vip.sangerbox.com/home.html).

### 2.8 Cell Culture and Treatment

Human umbilical vein endothelial cells (HUVECs) were purchased from Sciencell (USA, Cat. # 8000) and cultured in endothelial cell culture medium (ECM) (Sciencell, USA, Cat. # 1001) with 5% fetal bovine serum, 1% endothelial cell growth, and 1% antibiotic solution at 37 °C with 5% ${\mathrm{CO}}_{2}$. HUVECs from passages 3–5 were used in the experiments. HUVECs in the experimental groups were cultured in a sugar-free medium under hypoxic (1% $O_{2}$) conditions for 6 h and used in the subsequent experiments using the methodology established by Gabryel *et al*. [33]. All experiments were repeated in triplicate.

### 2.9 RNA Extraction and Quantitative Real-Time Polymerase Chain Reaction

We used the RNASimple Total RNA Kit (Cat. # DP419; Tiangen, China) to isolate the total RNA according to the manufacturer’s instructions. The cDNA was prepared using the Primescript RT Master Kit (RR036A; Takara, Japan) according to the manufacturer’s instructions, and then quantitative real-time polymerase chain reaction (qPCR) was carried out using the TB Green Premix Ex Taq™ II (RR820A; Takara, Japan). β-actin was used as the endogenous control; qPCR was performed on a QuantStudio3 Real-Time PCR System (Thermo scientific, USA). The relative expression of genes was calculated using the $2^{-\Delta \,\Delta \,\text{Ct}}$ method. The primer sequences are provided in Table 1. Relative gene expression is shown as mean ± SD. Student’s *t*-test was used to compare the relative expression differences between the groups.

**Table 1.** **Primer sequences for quantitative real-time PCR**.

Gene		Primer sequence
*ICAM1*	Forward	5′-AGCGGCTGACGTGTGCAGTAAT-3′
	Reverse	5′-TCTGAGACCTCTGGCTTCGTCA-3′
*VCAM1*	Forward	5′-GATTCTGTGCCCACAGTAAGGC-3′
	Reverse	5′-TGGTCACAGAGCCACCTTCTTG-3′
*GAS5*	Forward	5′-AAGCCTAACTCAAGCCATT-3′
	Reverse	5′-TTACCAGGAGCAGAACCA-3′
*H19*	Forward	5′-GACAGGAGAGCAGAGACT-3′
	Reverse	5′-GCAGCGAGACTCCAGGAA-3′
*MIR22HG*	Forward	5′-GAGCCGCAGTAGTTCTTC-3′
	Reverse	5′-TCAATCCAGCCAGTGTCT-3′
*LINC01091*	Forward	5′-GATCTGCTGTTAGAGGAGA-3′
	Reverse	5′-ATTTGCAGATGAAGTGATAC-3′
β *-actin*	Forward	5′-CACCATTGGCAATGAGCGGTTC-3′
	Reverse	5′-AGGTCTTTGCGGATGTCCACGT-3′

## 3. Results

### 3.1 Identification of DEGs between Aortic and LIMA PVAT of Patients with CHD

**Fig. 1.**
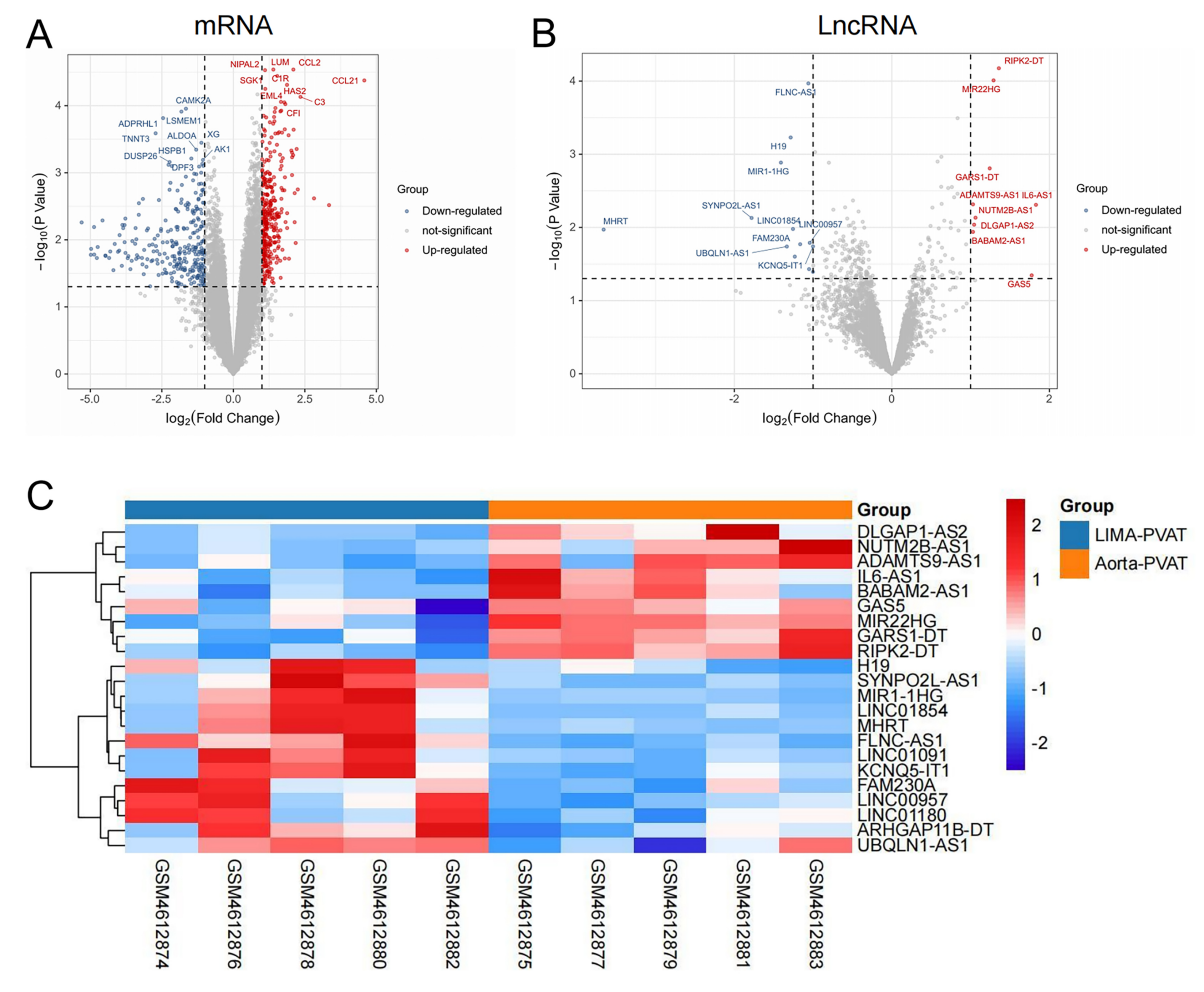
**Differentially expressed genes in aortic and left internal mammary artery (LIMA) perivascular adipose tissue (PVAT) in patients with coronary heart disease (CHD)**. (A) Volcano plots of differentially expressed mRNAs. (B) Volcano plots of differentially expressed lncRNAs. (C) Heatmap of differentially expressed lncRNAs. |$\log_{2}$FC| ≥1 and *p <* 0.05 were considered to indicate statistical significance.

The gene expression levels in the PVAT of five subjects with CHD were analyzed using a microarray; 643 genes were significantly differentially expressed between the proximal aorta and LIMA as a result (*p *< 0.05, |$\log_{2}$FC| ≥1). Among them, 352 were upregulated (343 mRNAs and 9 lncRNAs) (Fig. 1A,B) and 291 were downregulated (278 mRNAs and 13 lncRNAs) (Fig. 1A,B) in the aortic PVAT compared with those in the LIMA PVAT. The heatmap of all 22 differentially expressed lncRNAs is as follows (Fig. 1C).

### 3.2 Functional Enrichment Analysis of DEGs in Aortic PVAT Focuses on Immune and Inflammation-Related Pathways

**Fig. 2.**
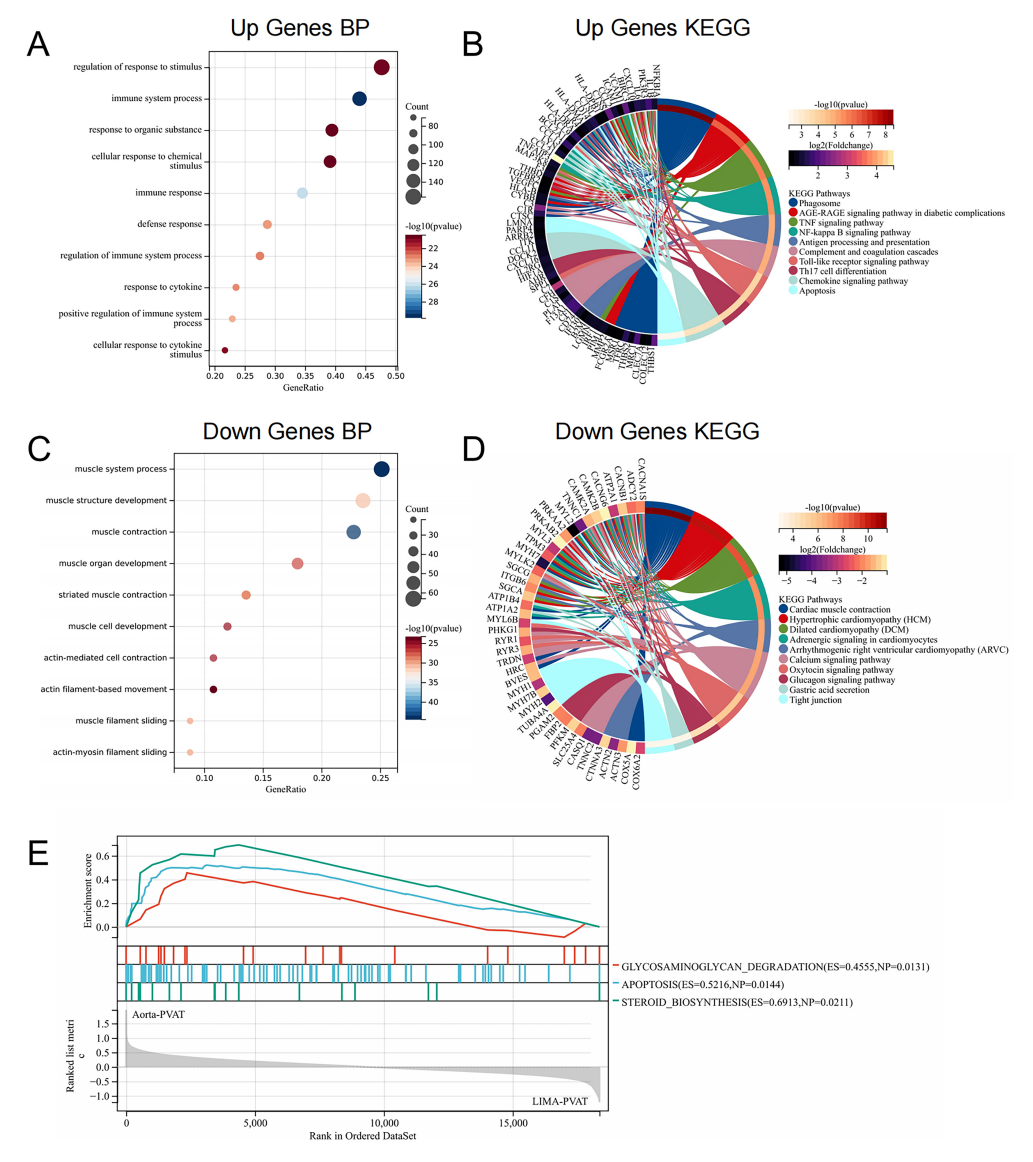
**Enrichment analysis of differentially expressed genes**. (A) Biological processes enriched by upregulated genes. (B) Kyoto Encyclopedia of Genes and Genomes (KEGG) pathway analysis of upregulated genes enrichment. (C) Biological processes enriched by downregulated genes. (D) KEGG pathways enriched by downregulated genes. (E) Upregulated GSEA in aortic perivascular adipose tissue (PVAT) in patients with CHD. Pathway enrichment analysis results with *p <* 0.05 after applying Benjamini–Hochberg correction were considered significant. A gene set was regarded as significantly enriched when *p <* 0.05 and false discovery rate (FDR) <0.25.

We then systematically assessed the potential biological functions of the DEGs using the GO term and KEGG pathway enrichment analyses. The following GO terms in the biological process (BP) category were enriched by the upregulated DEGs: immune system process, immune response, regulation of immune system processes, and response to cytokine (Fig. 2A). Consistent with these findings, the upregulated KEGG pathway enrichment analysis revealed that several immune-related pathways were enriched in aortic PVAT, including antigen processing and presentation, complement and coagulation cascades, and Th17 cell differentiation (Fig. 2B). In addition, several inflammation-related signaling pathways such as the tumor necrosis factor (TNF) signaling pathway, nuclear factor kappa-B (NF-κB) signaling pathway, and chemokine signaling pathway were enriched (Fig. 2B). The most downregulated BPs in aortic PVAT were primarily associated with muscle process and function (Fig. 2C). Similarly, the KEGG pathway analysis revealed several cardiomyopathy-related pathways that were significantly downregulated in aortic PVAT (Fig. 2D). In addition, apoptosis pathway and some metabolism-related pathways, such as glycosaminoglycan degradation and steroid biosynthesis were also significantly upregulated (Fig. 2E). Overall, these results suggest that genes within the aortic PVAT are involved in immunity, inflammation, muscle function, and metabolic processes.

### 3.3 ssGSEA Suggests that Patients with CHD Have a Higher Immune Cell Infiltration Score in Aortic PVAT

**Fig. 3.**
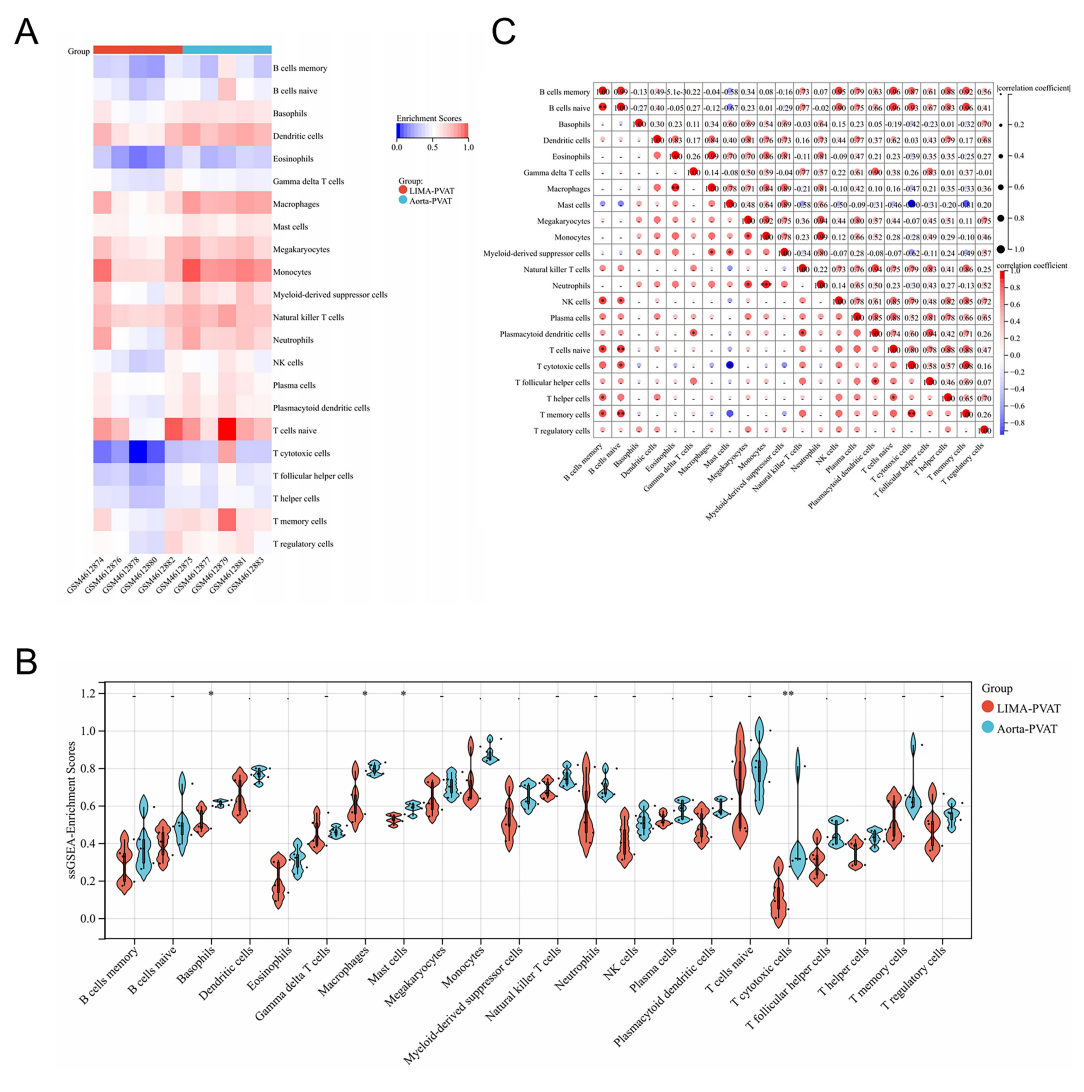
**Analysis of immune cell infiltration of perivascular adipose tissue (PVAT) in patients with coronary heart disease (CHD)**. (A) Heatmap of single-sample gene set enrichment analysis (ssGSEA) enrichment scores of ten samples. (B) Compositional differences of 22 immunocytes between aortic PVAT and LIMA PVAT. (C) Correlation matrix of 22 immunocytes proportions. The Wilcoxon test was used to evaluate the differences between the groups. The correlation between immune cell ssGSEA scores was calculated using Pearson’s method. * *p <* 0.05, ** *p <* 0.01, *** *p <* 0.001.

As the DEGs identified suggested that immune processes play an important role in PVAT dysregulation, we analyzed the infiltration level of immune cells in the PVAT of the aorta and LIMA using ssGSEA (Fig. 3). Notably, aortic PVAT had higher ssGSEA scores than LIMA PVAT. The ssGSEA scores of basophils, macrophages, mast cells, and cytotoxic T cells were significantly increased in aortic PVAT (Fig. 3B). Subsequently, we explored the potential relationships between the immunocytes using Pearson’s correlation analysis. Among all immunocytes, there was a strong correlation between ssGSEA scores of B and T cell subsets. There was a strong positive correlation between macrophages and eosinophils, a strong correlation between neutrophils and monocytes, and a negative correlation between cytotoxic T cells and mast cells (Fig. 3C).

### 3.4 Functional Co-Expression Network of Immune-Related lncRNAs

**Fig. 4.**
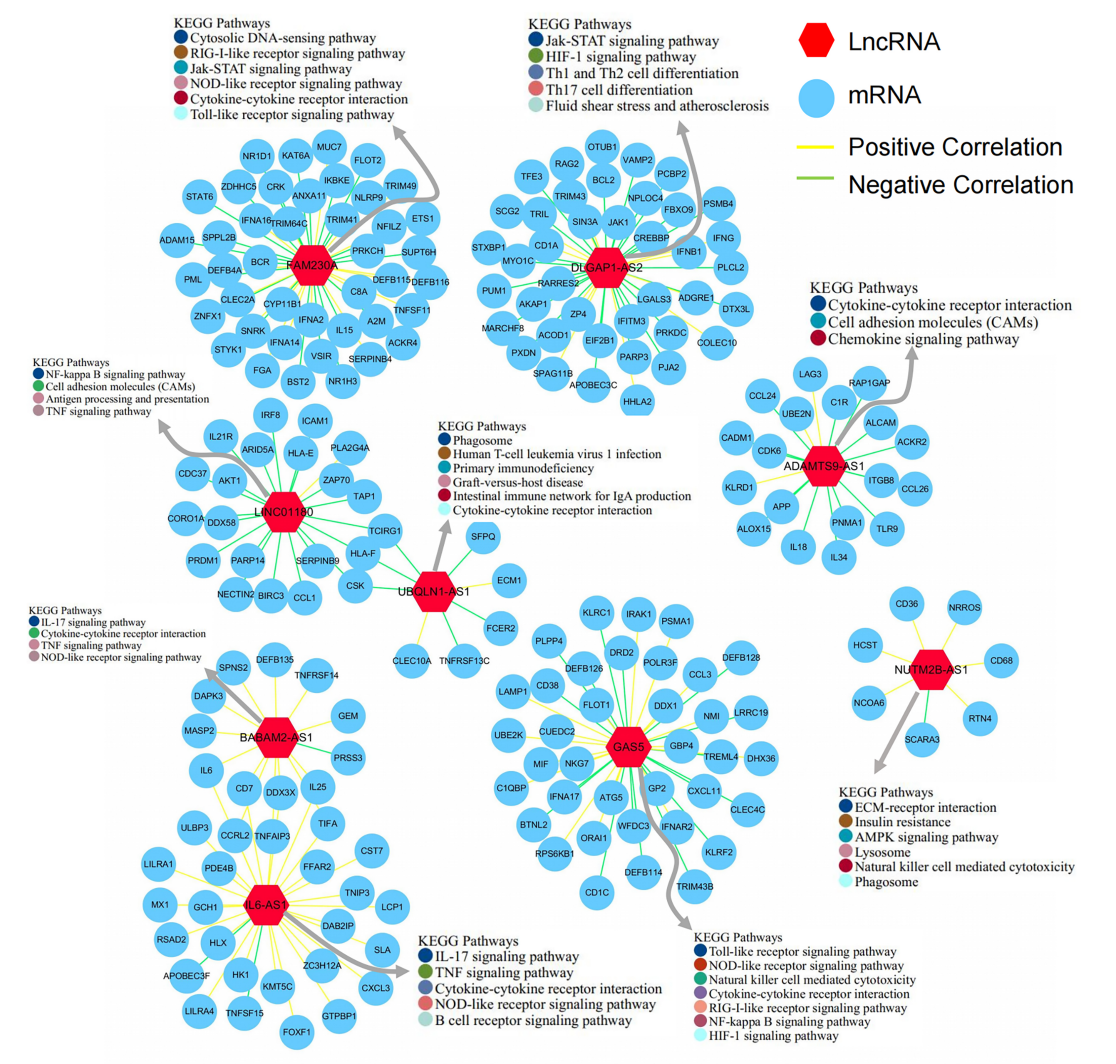
**Analysis of immune-related lncRNAs**. Co-expression network of lncRNAs associated with immune genes, and Kyoto Encyclopedia of Genes and Genomes (KEGG) pathway enrichment analyses of lncRNAs in the co-expression network. The KEGG pathway enrichment analysis results with *p <* 0.05 after applying Benjamini– Hochberg correction were considered significant.

The above results suggest that the immune process is very important, so we wondered whether these differentially expressed lncRNAs are also related to immunity. To screen lncRNAs that may be related to immunity, we first calculated the correlation coefficients between the expression of lncRNAs and immune-related genes in the chip. Subsequently, we selected lncRNAs with a high correlation coefficient to construct immune-related lncRNA networks (Fig. 4). We identified nine lncRNAs corresponding to immune-related genes. Thereafter, we determined the enriched KEGG pathways of the mRNAs that were strongly correlated with immune-related lncRNAs. The results showed that genes co-expressed with *FAM230A*, *UBQLN1-AS1*, and *BABAM2-AS1 *were mainly enriched in cytokine-cytokine receptor-related pathways, as well as inflammation and immune-related pathways. Genes co-expressed with *DLGAP-AS2 *were enriched in some pathways that regulate T cell differentiation, such as that of Th1, Th2, and Th17. The genes co-expressed with *LINC01180*, *ADAMTS9-AS1, *and *NUTMB-AS1 *were enriched in the inflammatory and cell adhesion pathways. The genes co-expressed with *IL6-AS1 *were enriched in the B cell receptor pathway in addition to some pathways related to inflammation or cytokines. The genes co-expressed with *GAS5 *were mainly enriched in cellular signaling pathways related to inflammation and hypoxic injury.

### 3.5 Prognosis Analysis of Immune-Related lncRNAs

**Fig. 5.**
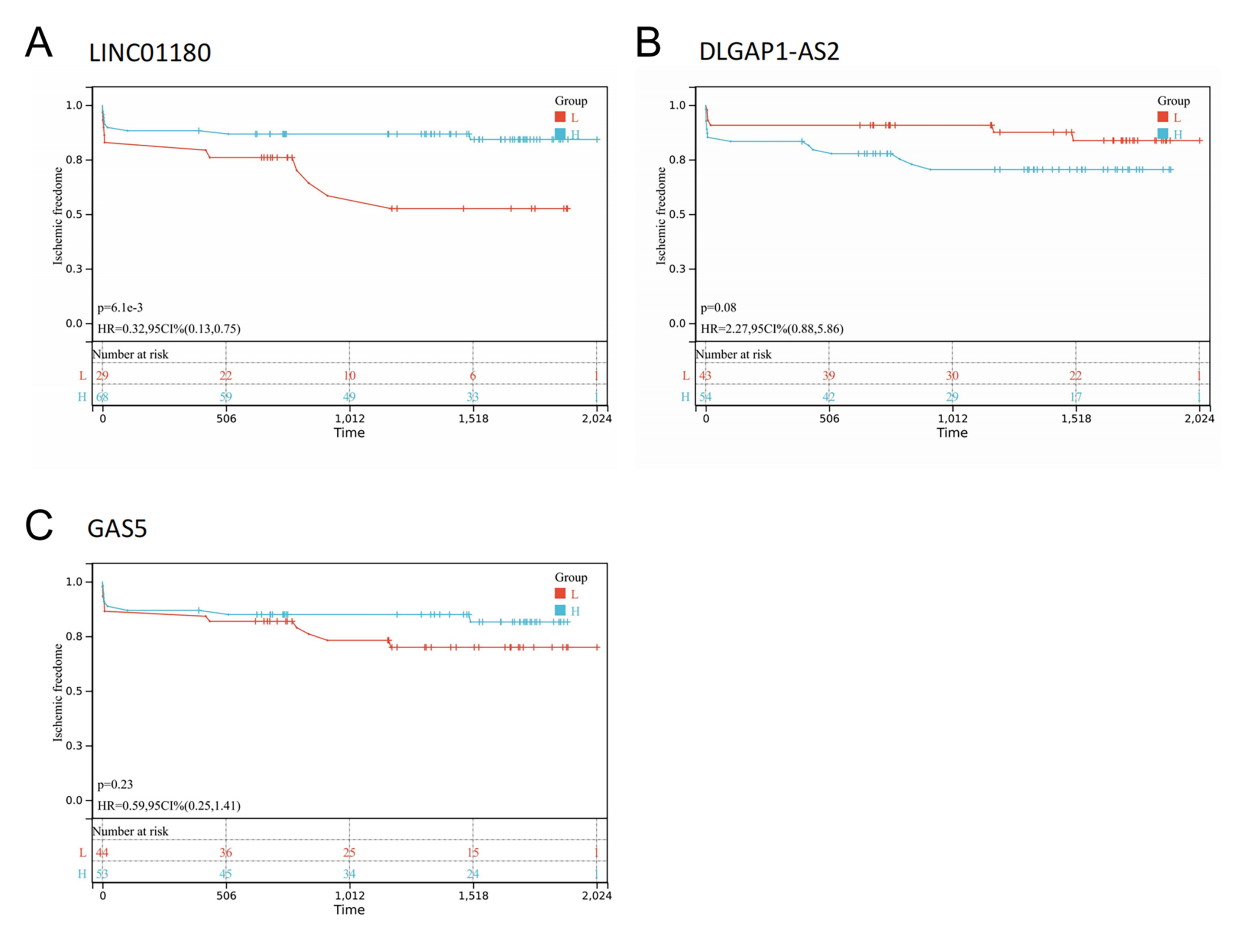
**Prognosis analysis of immune-related lncRNAs**. (A) Kaplan-Meier survival curves of *LINC01180*. (B) Kaplan-Meier survival curves of *DLGAP1-AS2*. (C) Kaplan-Meier survival curves of *GAS5*. The *p*-value was calculated using the log-rank test.

Subsequently, we analyzed the prognostic value for ischemic events of these immune-related lncRNAs in microarray data containing PBMCs of 97 patients with AS (Fig. 5). The results showed that *LINC01180 *may be a protective factor in AS (*p <* 0.05) (Fig. 5A), and *DLGAP1-AS2 *may be a risk factor for plaque instability, although the results were not statistically different (*p* = 0.08) (Fig. 5B).

### 3.6 Construction of lncRNA-Associated ceRNA Networks in Aortic PVAT

**Fig. 6.**
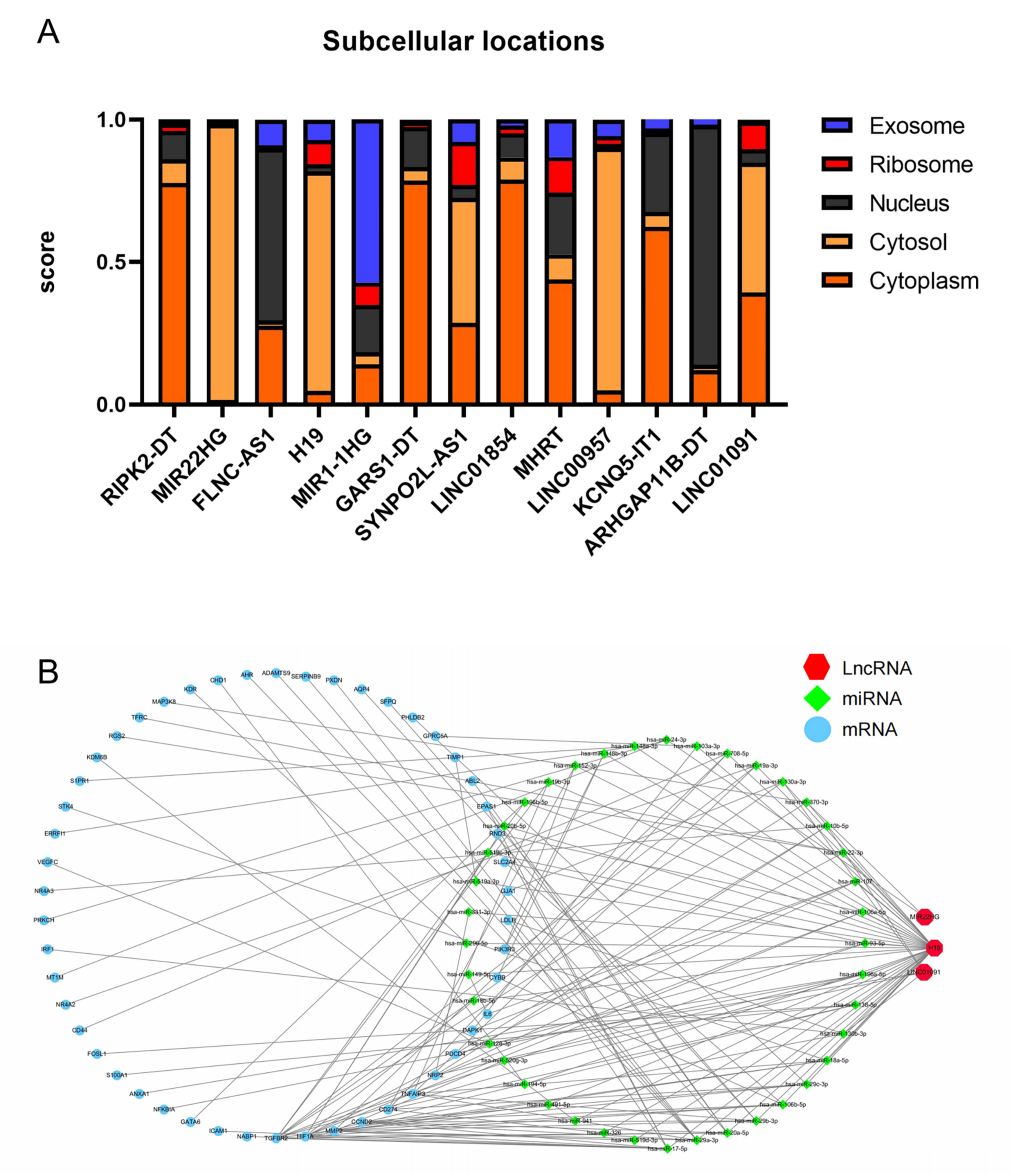
**Construction of a ceRNA network**. (A) Results of immune-unrelated lncRNAs’ cytoplasmic-nuclear localizations. (B) A lncRNA-associated ceRNA networks network.

As lncRNA functions are closely associated with their subcellular localization [34]. We investigated the positions of the remaining 13 lncRNAs. Database prediction results showed that *FLNC-AS1 *and *ARHGAP11B-DT *localized to the nucleus, *MIR1-1HG *localized to exosomes, and the others localized to the cytoplasm or cytosol (Fig. 6A). Based on these results, we hypothesized that these lncRNAs participate in CHD or AS progression by acting as ceRNAs. To test this hypothesis, we constructed a ceRNA network based on the starBase and miRTarBase databases, comprising 3 lncRNAs, 40 miRNAs, and 50 mRNAs (Fig. 6B).

### 3.7 Enrichment Analysis of Genes in ceRNA Network Focuses on Hypoxia and Inflammation-Related Signaling Pathway

**Fig. 7.**
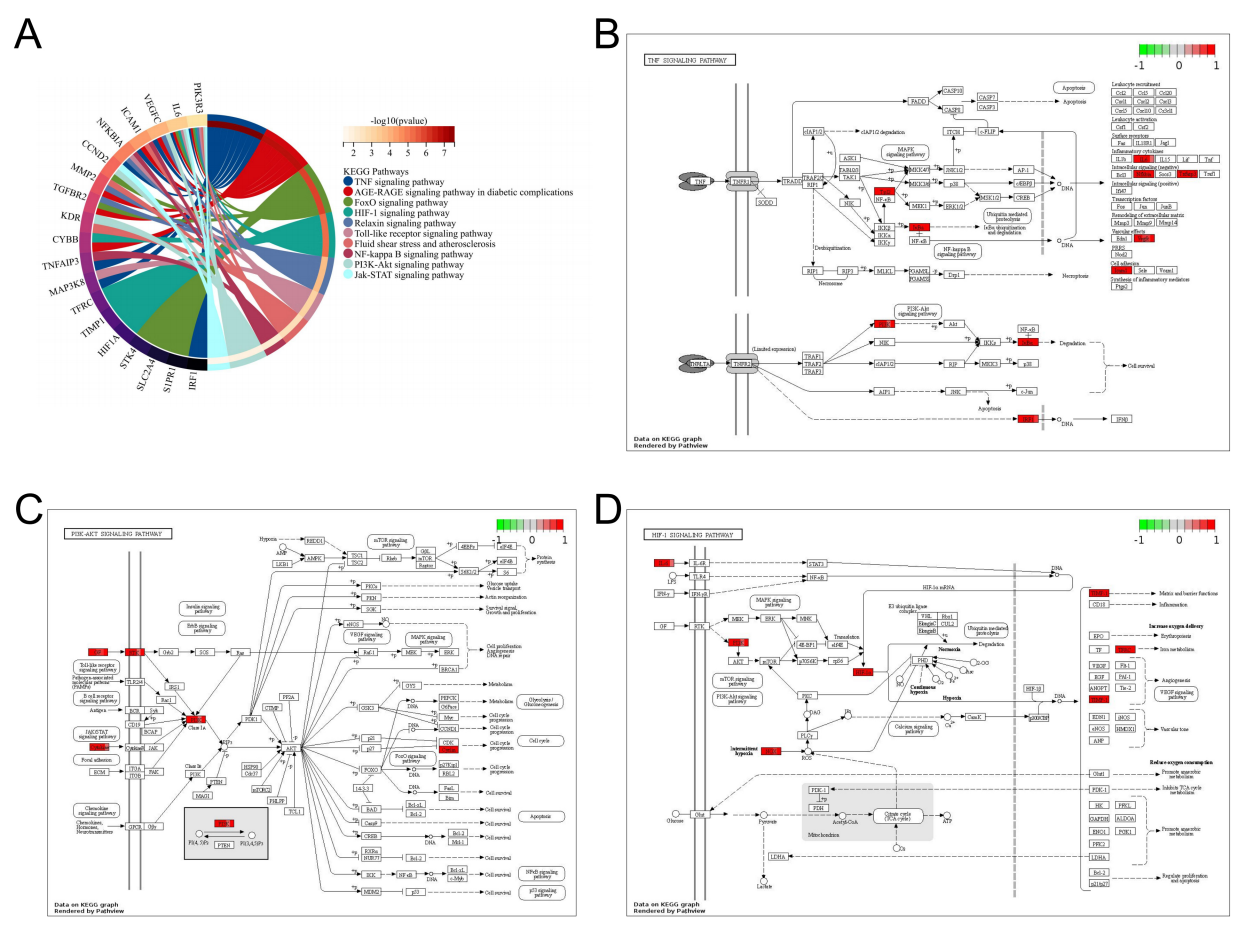
**Enrichment analysis of mRNAs in the ceRNA network**. (A) Kyoto Encyclopedia of Genes and Genomes (KEGG) pathway enrichment analysis of mRNAs in the ceRNA network. (B, C, D) Visualization of mRNAs present in the ceRNA network in the TNF, PI3K-Akt, and HIF1 signaling pathways. KEGG pathway enrichment analysis results with *p *< 0.05 after applying Benjamini–Hochberg correction were considered significant.

lncRNA-associated ceRNA networks can influence the regulation of the related mRNA-encoding genes. By constructing the ceRNA network, the functions of the lncRNAs can be inferred. The KEGG pathway enrichment analysis was performed on the genes in the identified networks, and several pathways were found to be significantly enriched (Fig. 7). The KEGG pathway enrichment analysis suggested that the TNF signaling pathway, hypoxia inducible factor-1 (HIF-1) signaling pathway, FoxO signaling pathway, PI3K-Akt signaling pathway, and other signaling pathways may associated with the occurrence and development of CHD and AS (Fig. 7A). Notably, IL-6 and PI3K participate in several of these signaling pathways, indicating that they may be potential therapeutic targets for patients with AS (Fig. 7B,C,D).

### 3.8 qPCR Verification of lncRNA Expression Associated with Hypoxia and Inflammation

**Fig. 8.**
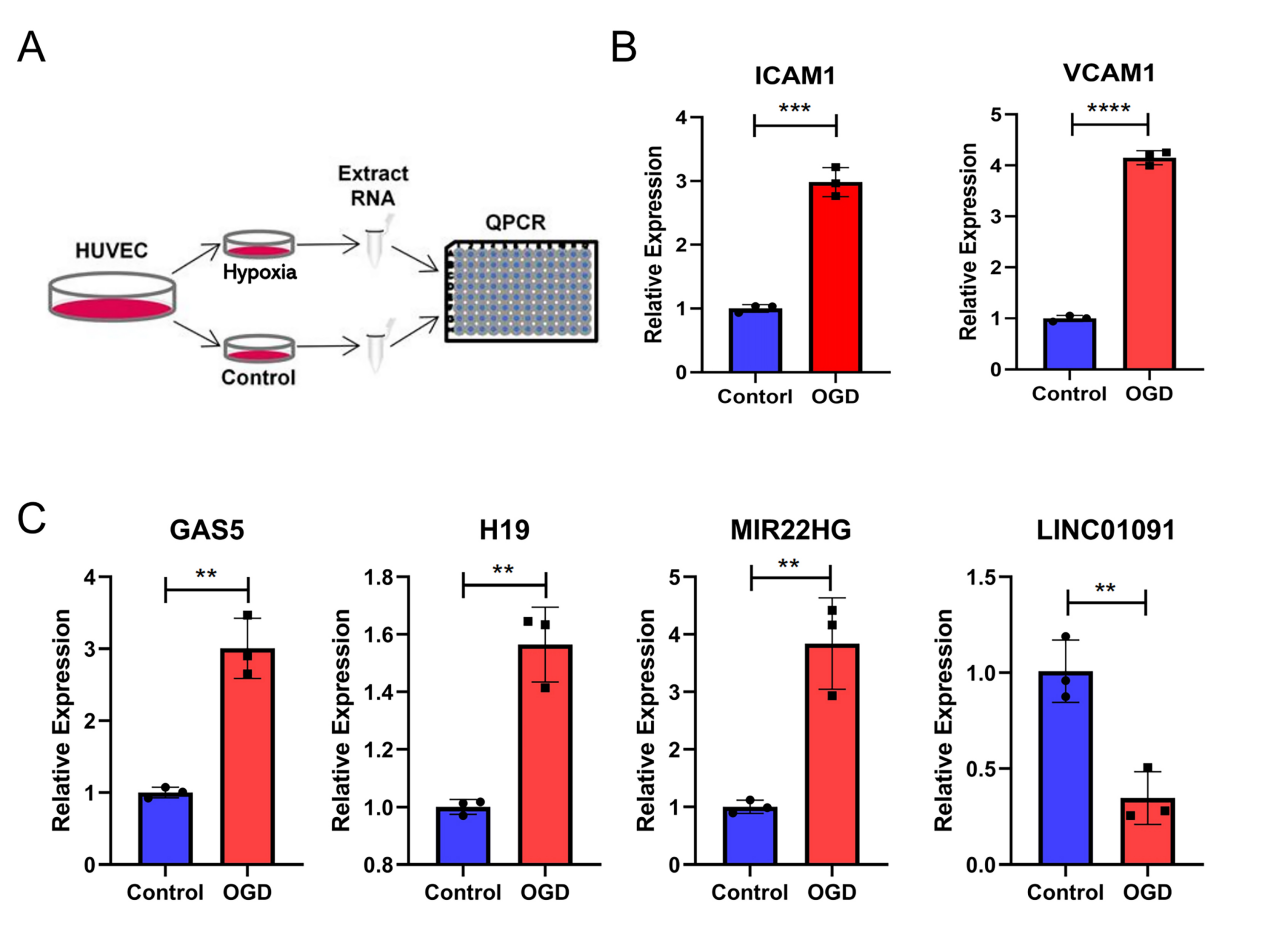
**Verification of lncRNA levels in hypoxia-treated endothelial cells by qPCR**. (A) The experimental design of this study. (B) The expression of *ICAM1 *and *VCAM1 *in human umbilical vein endothelial cells (HUVECs) with hypoxia treatment. (C) The expression of *GAS5*, *H19*, *MIR22HG,* and *LINC01091 *in HUVECs with hypoxia treatment. Student’s *t*-test was used to compare the relative expression differences between the groups. * *p *< 0.05, ** *p *< 0.01, *** *p *< 0.001.

Finally, we used qPCR to validate the levels of the lncRNAs selected above (Fig. 8). *ICAM1 *and *VCAM1 *were significantly upregulated in HUVECs after hypoxia treatment (Fig. 8B). The results showed that *GAS5*, *H19*, and *MIR22HG *were upregulated in HUVECs after hypoxia treatment, whereas *LINC01091 *was downregulated (Fig. 8C).

## 4. Discussion

Based on the chip data published by Mazzotta *et al*. [13], we did a more in-depth analysis. According to Mazzotta *et al*. [13], aortic PVAT from patients with CHD shows a higher degree of inflammation than LIMA PVAT, with higher levels of inflammatory factors such as IL-1, MCP-1, and IL-6, which positively correlate with the Gensini score. Our results showed that aortic PVAT in patients with CHD might be accompanied by higher immune cell infiltration. The expression of nine lncRNAs strongly correlated with the expression of immune-related genes, including *FAM230A*, *NUTM2B-AS1*, *GAS5*, *ADAMTS9-AS1*, *BABAM2-AS1*, *DLGAP1-AS2*, *IL6-AS1*, *LINC01180*, and *UBQLN1-AS1*. The differential expression of *H19*, *LINC01091*, and *MIR22HG *in patients with CHD may be related to oxidative stress and hypoxia. Hence, the aforementioned lncRNAs may be associated with AS and CHD and should be further investigated.

Recently, with the application of flow cytometry, immunofluorescence staining, and single-cell sequencing, our understanding of the diversity of immune cells in PVAT has considerably improved. As listed in Table 2 (Ref. [35, 36, 37, 38, 39, 40, 41]), PVAT mainly consists of T cells, B cells, NK cells, macrophages, neutrophils, dendritic cells, eosinophils, and mast cells [35, 36, 37, 38, 39, 40, 41]. Numerous studies have demonstrated that PVAT secretes various cytokines and plays an important role in multiple cardiovascular pathophysiological processes by inducing immune cell infiltration or directly affecting smooth muscle cells and vascular endothelial cells [9, 42]. Srikakulapu *et al*. [36] discovered that most B cells in and around the aorta are derived from PVAT, with a substantial proportion of these B cells belonging to the B-1 subgroup, and that local IgM production may play a role in AS protection. Numaguchi *et al*. [43] compared PVAT around the coronary artery (CA-PVAT) and the internal thoracic artery (ITA-PVAT) and observed that M1 macrophages increased more in CA-PVAT than in ITA-PVAT, whereas the ratio of M2 to M1 macrophages decreased. Therefore, it is necessary to explore the infiltration of immune cells in PVAT in disease states.

**Table 2.** **Experimentally validated types of immune cells in PVAT**.

Author	Immune cell type	Sample source	Method	References
Farias-Itao *et al*.	B cells; T cells; macrophages	Human	immunohistochemistry	[35]
Srikakulapu *et al*.	B cells; T cells	Human; Mouse	Flow cytometry	[36]
Withers *et al*.	Eosinophils	Mouse	Flow cytometry	[37]
Sagan *et al*.	B cells; T cells; Macrophages; Granulocytes; NK cells; Dendritic cells	Human	Flow cytometry	[38]
Kumar *et al*.	B cells; T cells; Macrophages; NK cells; Mast cells; Neutrophils	Rats	Flow cytometry	[39]
Nosalski *et al*.	B cells; T cells; Macrophages; NK cells	Rats	Flow cytometry	[40]
da Silva *et al*.	B cells; T cells; M1 Macrophages; M2 Macrophages	Mouse	Flow cytometry	[41]

The score of immune cells in each sample can be calculated by combining chip data and bioinformatic analysis methods [44]. We evaluated the infiltration levels of immune cells using the ssGSEA and revealed significant differences (*p <* 0.05) in the infiltration levels of four types of immune cells. Although there was no statistical difference in the scores of other types of immune cells, the immune cell score of aorta PVAT was higher overall, and there was also a strong correlation between the scores of different types of immune cells, indicating that aorta PVAT immune processes may be more active. The role of macrophages and killer T cells in promoting the development of AS has been reported and confirmed by many studies, and the higher infiltration levels of macrophages and killer T cells in aorta PVAT may be an AS risk factors. Basophils and mast cells are two distinct cell types with similar activation mechanisms that play causative roles in allergic, inflammatory, and autoimmune diseases [45]. Among these two types of cells, mast cells have been studied more in cardiovascular diseases, and the mechanism of its action is relatively clear. Studies have shown that mast cell degranulation leads to the release of multiple fibrotic mediators, leading to cardiac fibrosis, and that mast cells can also contribute to plaque formation by stimulating foam cell formation and inflammation in the local environment [46, 47, 48]. Compared with mast cells, basophils are present in lower numbers in the body, and their relationship with cardiovascular disease is not clear. Pizzolo *et al*. [49] evaluated the association between white blood cell count and mortality in 823 patients with contrast-proven and clinically stable CHD; they identified that high levels of neutrophils and basophils Blood counts can predict mortality in patients with clinically stable CHD, and that these mechanisms may be related to thrombotic. Therefore, the specific mechanism of action of basophils in cardiovascular diseases is worthy of further study and exploration.

In addition to immunity and inflammation, epigenetics plays an important role in AS [50]. In our study, we constructed a co-expression network to identify lncRNAs that may be related to immunity. Yi *et al*. [51] revealed that *IL6-AS1 *is upregulated in chronic obstructive pulmonary disease and promotes the expression of inflammatory factors, particularly IL-6. This is consistent with the results obtained from our analysis that *IL6-AS1 *may affect the secretion of IL-6 through the TNF signaling pathway (Fig. 4). *GAS5 *has also been studied extensively, and some of these studies have also shown that *GAS5 *is involved in the regulatory process of immune cells, and thus affect disease progression. Ahmad *et al*. [52] found that GAS5 plays a key role in macrophage differentiation, polarization, and regulation of innate functions including antigen processing and phagocytosis. Li *et al*. [53] found that *GAS5 *inhibited Th17 differentiation by promoting TRAF6 mediated ubiquitination of STAT3, thereby alleviating immune thrombocytopenia. These lncRNAs exhibit functions well beyond the regulation of immune processes. In addition to being related to immunity, *GAS5 *has also been reported to regulate cellular inflammation, lipid uptake, and other processes [54, 55, 56]. In the data set GSE21545, we found that LINC01180 was associated with the instability of AS plaques through univariate Cox regression analysis. The high expression of LINC01180 in PBMCs of patients with AS seems to have a protective effect and should be explored in future studies. Therefore, our findings can provide a foundation for future research on the functions and downstream mechanisms of these lncRNAs.

In recent years, the role of several lncRNA–miRNA–mRNA axes are thought to play a role in the pathogenesis of cardiovascular diseases [57, 58, 59]. In this study, we constructed a lncRNA-associated ceRNA network. The KEGG pathway enrichment analyses of the protein-coding genes in the network revealed that they are primarily involved in response to hypoxia and oxidative stress (Fig. 7A). Ramirez *et al*. [10], have demonstrated that although adipocytes are primarily present in the PVAT, infiltrating immune cells may become more important under hypoxic and inflammatory conditions. The HIF signaling pathway is the most used for cells to respond to hypoxia, studies have shown that this signaling pathway is involved in the development of the heart, AS and myocardial ischemia [60]. In endothelial cells, smooth muscle cells, macrophages, and other cells, the HIF signaling pathway plays an important regulatory role in hypoxic injury [60]. The janus kinase-signal transducer and activator of transcription (JAK-STAT) signaling pathway is the alternative pathway of the second messenger system, through which chemical signals from outside the cell are transmitted to the cell to induce DNA transcription and intracellular activity, and involved in regulating inflammation [61]. The PI3K/Akt signaling pathway is also closely related to endothelial cell dysfunction and AS [62]. Therefore, lncRNAs in the ceRNA network may participate in the regulation of cardiovascular disease by regulating intracellular inflammation and hypoxia response, which should be studied further. Our results further illustrate the important role of PVAT in the occurrence and development of CHD and AS.

AS is a complex pathological process, and its pathogenesis remains unclear, clinicopathological examinations show that endothelial cells in CVD patients have severe structural and functional damage, indicating that vascular endothelial cells play an important role in the occurrence and development of AS [63]. Therefore, we verified the expression of these lncRNAs related to inflammation and hypoxia by constructing a model of hypoxia endothelial cell injury. A previous study revealed that the expression of *GAS5 *was increased in ox-LDL-treated THP-1 cells and that *GAS5 *knockout could potentially promote the reverse transport of cholesterol, inhibit intracellular lipid accumulation, and prevent AS progression [55]. Li *et al*. [64] revealed that the expression of *GAS5 *increased in atherosclerotic mouse plaques, and *GAS5 *knockout promoted endothelial cell proliferation, reduced apoptosis, and improved AS. Chen *et al*. [65] revealed that *GAS5 *is related to the aging of smooth muscle and plays a role in the pathological process of AS. *H19*, a lncRNA that was among the first to be discovered and studied, has also been reported to be involved in cardiovascular diseases, CHD, and AS. Zhang *et al*. [66] showed that plasma *H19* was elevated in patients with CHD, and its level could serve as an independent predictor of CAD. Hobuss *et al*. [67] found that *H19* is upregulated in the acute phase after cardiac ischemia in mice, and *in vitro* cellular experiments found that hypoxia leads to upregulation of *H19* in several cardiac cell types. Cao *et al*. [68] found that *H19* expression was elevated in human atherosclerotic plaques and oxidized-LDL (ox-LDL) treated HUVECs, and that *H19* knockdown inhibited ox-LDL induced HUVEC inflammation, apoptosis, and oxidative stress.

Taken together, our findings suggest an important regulatory role for *GAS5* and *H19* in AS and CHD, some of which were mechanistically explored in endothelial cells. Our findings are consistent with previously published studies, *GAS5* and *H19* expression were found to be elevated in the endothelial cell hypoxia injury model. In addition, the relationship among *MIR22HG* and *LINC01091* and cardiovascular and endothelial cells has not been reported in the literature. *MIR22HG *has been reported to be dysregulated in various tumors [69]. However, the relationship between *MIR22HG *and cardiovascular diseases is unclear. A previous study reported that the expression of *MIR22HG *is upregulated in the hypoxic myocardium, thus aggravating hypoxic injury of cardiomyocytes through NF-κB activation [70]. The relationship between *LINC01091 *and cardiovascular diseases remains unknown. In summary, the relationship between *GAS5 *and *H19 *and cardiovascular disease is relatively clear, but the relationship among *MIR22HG*, *LINC01091*, and cardiovascular disease requires further study.

Although lncRNAs have been discovered to be significant regulators of cardiovascular disease, our understanding of the role of these molecules in cardiovascular disease has not yet been extensively investigated [71, 72]. In this study, Bioinformatics was used to investigate the differentially expressed lncRNAs in CHD patients, and to speculate their roles. Some of these lncRNAs, such as *GAS5*, *H19*, and *IL6-AS1*, were compatible with earlier research, indicating that our bioinformatics approach yielded relatively credible results. Finally, our results can provide research ideas for lncRNA studies in AS and CHD. Inevitably, our study has some limitations. First, the number of samples of the source chip was relatively small; therefore, follow-up experimental verification is necessary. Second, the control group was the LIMA PVAT of the same patient instead of the PVAT of a normal human aorta. If the PVAT of a normal human aorta can be obtained as a control, more information may be obtained; however, as stated by the contributor of the chip, due to the particularity of this tissue, it is difficult to obtain it only by performing thoracotomy. LIMA is the blood vessel often selected for coronary artery bypass surgery. Therefore, comparing the aortic PVAT and LIMA PVTA could also partially address the problem. Finally, many of our analyses were based on bioinformatic methods. Some of the lncRNAs mentioned above that may be related to immunity are obtained based on bioinformatics analysis without experimental support and, therefore, need to be verified by flow cytometry or single-cell sequencing and experiments.

## 5. Conclusions

In conclusion, through a comprehensive analysis of lncRNAs differentially expressed across different PVAT in patients with CHD, we characterized several lncRNAs that may be related to immunity. We also constructed lncRNA-associated ceRNA networks. These findings further expand our understanding of the pathogenesis of CHD and AS and provide new insights into the function of PVAT in patients with AS and CHD.

## Abbreviations

CHD, Coronary heart disease; AS, Atherosclerosis; lncRNA, long non-coding RNA; ceRNA, competing endogenous RNA; BP, Biological Process; DEG, Differentially expressed genes; ECM, Endothelial cell culture medium; FDR, False discovery rate; GEO, Gene Expression Omnibus; GO, Gene Ontology; HUVEC, Human umbilical vein endothelial cells; Qpcr, Quantitative real-time polymerase chain reaction; IMA, Internal mammary artery; KEGG, Kyoto Encyclopedia of Genes and Genomes; LIMA, Left internal mammary artery; PVAT, Perivascular adipose tissue.

## Supplementary Material

Supplementary material associated with this article can be found, in the online version, at https://doi.org/10.31083/j.rcm2310341.
