# Supplementary material for: Comprehensive Analysis of Differentially Expressed lncRNAs in the Perivascular Adipose Tissue of Patients with Coronary Heart Disease
Source: Rev Cardiovasc Med. 2022 Oct 11;23(10):341. doi: 10.31083/j.rcm2310341 (PMC11267359; doi:10.31083/j.rcm2310341)
Supplement: Supplementary file 1 [file 2153-8174-23-10-341-s1.docx]

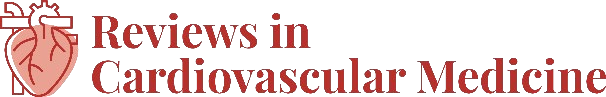
Comprehensive Analysis Differentially Expressed lncRNAs in the Perivascular Adipose Tissue of Patients with Coronary Heart Disease

**Supplementary Material**

Supplementary Table 1. Clinical features of included CHD patients.

| Clinical features of included patients | n=5 |
| --- | --- |
| Age (years) | 60±5 |
| Sex (Male, %) | 100 |
| Obese (%) | 80 |
| LDL cholesterol (mg/dl) | 89±31 |
| Statin medication use (%) | 100 |

Data are mean ± SD.

Supplementary Table 2. Clinical features of 97 patients with atherosclerosis.

| Clinical features of 97 patients | n=97 |
| --- | --- |
| Age (years) | 70.8 ± 9.38 |
| Sex (Female, %) | 25.8% |
| Body mass index (kg/m^2^) | 26.5 ± 4.15 |
| LDL (mmol/L) | 2.46 ± 0.97 |
| Current or previous smoker | 100 |
| Type 2 diabetes mellitus (%) | 26.8% |
| Follow-up time (d) | 1,159 ± 631 |
| Ischemic event during follow-up (%) | 21.6% |

Data are mean ± SD
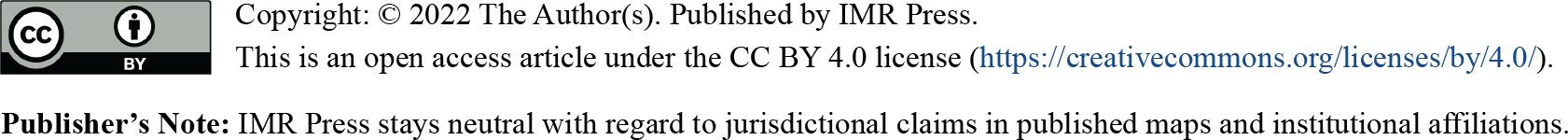
.
